# Helicobacter pylori Resistance to Triple Therapy in a Multicultural Population in New York City

**DOI:** 10.7759/cureus.19883

**Published:** 2021-11-25

**Authors:** Silvia Fernandez-Perez, Gilberto Velazco, Joanna M Lenik, Yvette Achuo-Egbe, Jennifer Harley

**Affiliations:** 1 Gastroenterology and Hepatology, New York Medical College (NYMC) - Metropolitan Hospital Center, New York, USA; 2 Internal Medicine, New York Medical College (NYMC) - Metropolitan Hospital Center, New York, USA

**Keywords:** h. pylori, antibiotic resistance (abr), drug susceptibility testing and antibiotic resistance, bacterial resistance, antimicrobial resistance, helicobacter pylori

## Abstract

Introduction: *Helicobacter pylori* infection (HPI) has become a worldwide concern due to its associations with intestinal and extraintestinal disease including cancer, autoimmune phenomena, and vitamin deficiencies. HPI has been found to affect Hispanics at higher rates compared with non-Hispanics in the USA. Hispanics comprise most of the patient population at Metropolitan Hospital in New York City. Growing concerns about antibiotic resistance led to the reconciliation of treatment guidelines with the consensus of bismuth quadruple therapy as the first-line treatment, replacing clarithromycin-based triple therapy. We conducted a retrospective study to explore the resistance rate of *Helicobacter pylori *to triple therapy in patients at Metropolitan Hospital.

Objective: To explore the resistance rates of *Helicobacter pylori* in infected patients treated with clarithromycin-based triple therapy in Metropolitan Hospital over a five-year period.

Materials and Methods: Charts of all patients who underwent upper endoscopy during a five-year period were retrospectively reviewed. Overall, 2000 patients were screened for presence of HPI. We included 322 patients with a demonstrated HPI obtained from biopsies taken during upper endoscopy within the study period. Inclusion criteria were patients older than 18 years old with positive HPI who were prescribed therapy. Exclusion criteria were patients with positive HPI who did not receive treatment for the infection and patients without a confirmatory diagnosis of infection. We further reported on three groups based on the implemented therapy. Each treated group was divided into three subgroups based on eradication testing. Treatment compliance was documented. The patient population was demographically characterized by ethnicity, age at diagnosis, body mass index (BMI), and sex.

Results: Of the 322 patients included in the study, 258 were Hispanics (80%). The eradication rate among patients treated with selected clarithromycin-based therapies was found to be statistically significant when compared with other HPI therapies. There was no statistically significant difference between the studied group with respect to age, sex, ethnicity, and BMI. In the group of patients with suspected clarithromycin resistance, antimicrobial sensitivity testing was ordered in one case.

Discussion: HPI varies with race and ethnicity. Within the USA, the prevalence is lowest among non-Hispanics. Ethnicity and age, sex, and BMI were not factors that impacted treatment outcomes. We found that triple therapy with a proton pump inhibitor, amoxicillin, and clarithromycin (PAC) was used as a first-line treatment, consistently showing a low rate of resistance. The eradication rate among patients treated with PAC was found to be statistically significant when compared with all other therapies. It is significant for the hospitals with limited resources, where initial treatment follows the “test-and-treat” strategy. Quadruple therapy as the first-line treatment raises concerns about medication costs, insurance coverage, side effects, and dosing, which may have a significant impact on patient compliance.

Conclusion: Our study showed that selected clarithromycin-based therapies were superior for HPI eradication when compared with non-clarithromycin-based triple therapy in low-resistance communities. Culture with antimicrobial susceptibility testing was used in a de minimis number of cases, which raises awareness for future study.

## Introduction

*Helicobacter pylori* infection (HPI) has become a worldwide concern given its association with gastric cancer including intestinal mucosa-associated lymphoid tissue (MALT) lymphoma and adenocarcinoma, dyspepsia, and other extraintestinal diseases, such as iron deficiency anemia, idiopathic thrombocytopenic purpura, and vitamin B12 deficiency [1]. This infection has been found to affect Hispanics living in the USA at higher rates as compared with non-Hispanics (Hispanic: 60%; Black: 50%; White: 25%) [2].

Hispanics comprise the majority of the patient population at Metropolitan Hospital in New York City (more than 60%), and HPI is a common reason for an office visit in this area with unknown rates of clarithromycin resistance [3]. Increasing rates of resistance of *Helicobacter pylori *to clarithromycin-based therapy were found in studies performed in different geographic regions outside of the USA.

Growing concerns about antibiotic resistance led to the reconciliation of previously issued international treatment guidelines. As a result, bismuth quadruple therapy (proton pump inhibitor, bismuth, metronidazole, and tetracycline) is now recommended by the Canadian Association of Gastroenterology/Canadian Helicobacter Study Group (Toronto Consensus), the American College of Gastroenterology (ACG), and the European Helicobacter and Microbiota Study Group (Maastricht V/Florence Report) as the first-line treatment, replacing the previously used clarithromycin-based triple therapy (proton pump inhibitor, amoxicillin, and clarithromycin (PAC)), with exceptions made by the ACG and the European Study Group for populations or regions with well-studied and established low clarithromycin resistance (<15%) with no prior macrolide exposure or when microbial sensitivity is performed prior to therapy initiation [4-6].

We conducted a retrospective study to explore the resistance rate of *Helicobacter pylori* to triple therapy in treated patients at Metropolitan Hospital.

## Materials and methods

Using the QuadraMed and endoPRO software for appropriate data collection, a retrospective chart review of electronic medical records of all patients who underwent esophagogastroduodenoscopy (EGD) during a five-year period from July 2014 to July 2019 was conducted.

**Figure 1:**
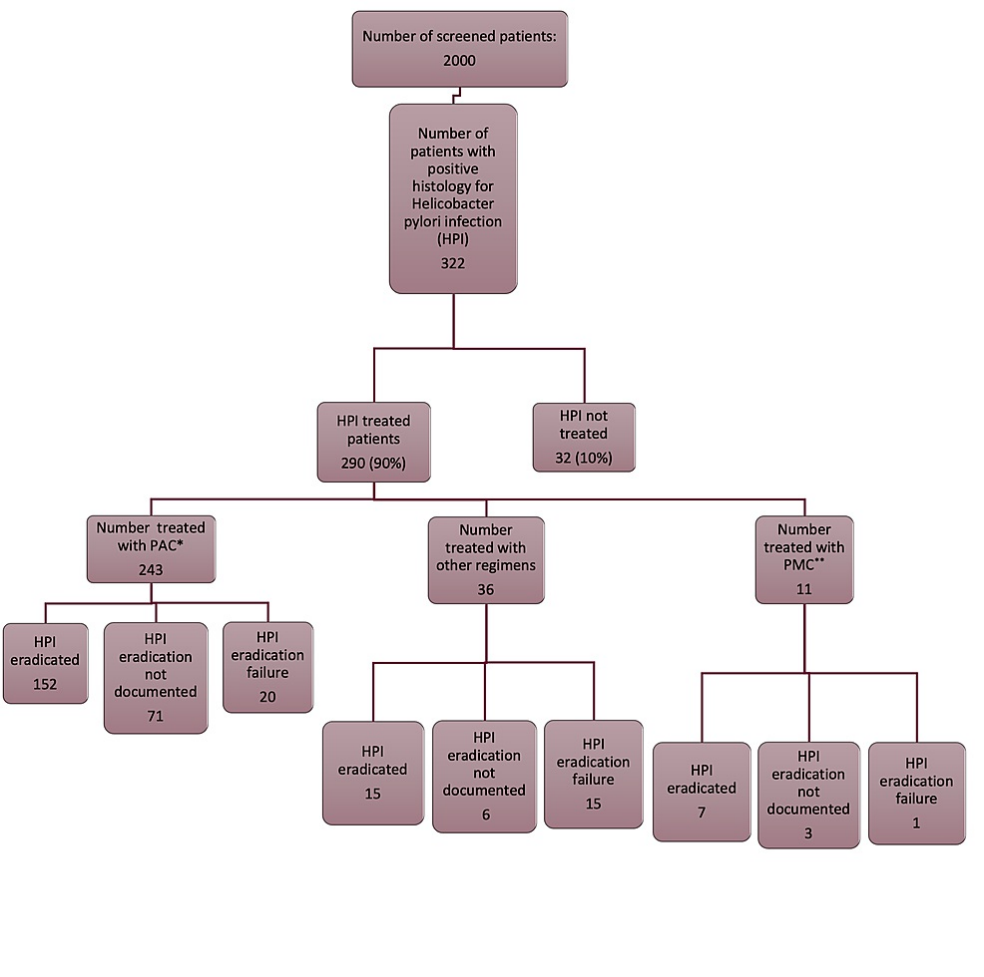
Helicobacter pylori infection data. *PAC: proton pump inhibitor plus amoxicillin plus clarithromycin **PMC: proton pump inhibitor plus metronidazole plus clarithromycin

A total of 2000 patients were screened for presence of HPI, and 322 patients were included in the study. The studied group comprised of all patients with a demonstrated HPI obtained from biopsies taken during EGD within the study period during phase one of data gathering. During the second phase of data gathering, we reported on three groups that included patients treated with a proton pump inhibitor plus amoxicillin plus clarithromycin (PAC), patients treated with a proton pump inhibitor plus metronidazole plus clarithromycin (PMC), and patients receiving other therapies. Each treated group was divided into three subgroups based on eradication testing comprising of patients with HPI eradication after treatment, patients with persistent HPI after treatment, and patients not tested for eradication. Data selection is detailed in Figure 1.

Eradication was confirmed by repeated endoscopic biopsies or non-endoscopic testing such as the *Helicobacter pylori* stool antigen test.

Documentation of treatment compliance, including duration, treatment interruptions, and appropriate dosing, was recorded for those patients with persistent HPI. Infected patients who were lost to follow-up and presented after a significant lapse of time with positive HPI results were classified as persistent infections due to eradication failure.

Furthermore, the study population was demographically characterized by the following variables: ethnicity, age at diagnosis, body mass index (BMI), and sex.

The inclusion criteria were patients older than 18 years of age with positive HPI confirmed on histopathology who were prescribed therapy at Metropolitan Hospital. The exclusion criteria were patients with positive HPI documented by histopathology who did not start any treatment and patients who did not have a confirmed diagnosis of infection on histopathology.

Analysis including percentage distribution was used for the interpretation of the collected data. Uncorrected chi-square analysis was used for the calculation of the p-value.

## Results

**Figure 2:**
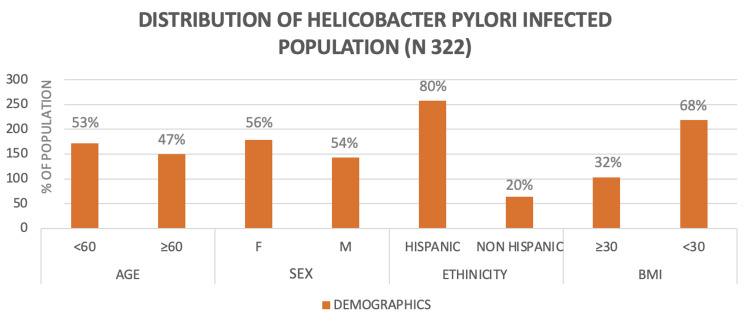
Characterization of the Helicobacter pylori-infected population.

Of the 322 patients included in the study, 258 were of Hispanic ethnicity (80%). The characterization of the population is detailed in Figure 2. 

The eradication rate among patients treated with PAC was found to be statistically significant when compared with all other therapies (Table 1).

**Table 1:** Eradication of HPI with PAC versus other non-clarithromycin-based regimens.

	PAC	Other therapies	P-value
Eradication achieved	152	15	<0.031
Eradication failed	20	6	

Comparison between groups treated with clarithromycin-based therapies, including PAC and PMC, did not show statistical significance (Table 2).

**Table 2:** Eradication of HPI with first-line PAC versus PMC.

	PAC	PMC	P-value
Eradication achieved	152	7	0.94
Eradication failed	20	1	

There was no statistically significant difference between the studied groups with respect to age, sex, ethnicity, and BMI (Table 3).

**Table 3:** Demographics and eradication rates of HPI with PAC.

Demographics		Eradication achieved (N, %)		Eradication failed (N, %)		P-value
Age	<60	78	51.3	11	55	0.75
	≥60	74	48.6	9	45	
Sex	Female	87	57.2	15	75	0.12
	Male	65	42.7	5	25	
Ethnicity	Hispanic	123	80.9	19	95	0.11
	Non-Hispanic	29	19.1	1	5	
BMI	≥30	51	33.5	5	25	0.44
	<30	101	66.4	15	75	

In the group of patients with suspected clarithromycin resistance, antimicrobial sensitivity testing was performed in one case.

## Discussion

After 2000 patients were screened for this retrospective study, we found that the vast majority of patients who were classified for the second phase were Hispanic. HPI varies with race and ethnicity. In general, within the USA, the prevalence is lower among non-Hispanic Whites than among other ethnic groups [5]. Although our patient population at Metropolitan Hospital in New York City is predominantly Hispanic and African American, our study concluded that 80% of the affected patients were Hispanic. Notably, our data suggest that ethnicity and age, sex, and BMI were not factors that impacted treatment outcomes.

During the period from July 2014 to July 2019, we found that triple PAC was used as a first-line treatment consistently. Of those treated patients, the overwhelming majority had a documented cure, showing a low rate of resistance for triple therapy. In this retrospective study, the eradication rate among patients treated with PAC was found to be statistically significant when compared with all other therapies combined. Other therapies used included PMC and bismuth-based therapies. This suggests that triple therapy with PAC was a cost-effective regimen in this low clarithromycin-resistant community. Our study showed that selected clarithromycin-based therapy such as PAC was statistically significant for HPI eradication in our study population when compared with non-clarithromycin-based triple therapies. This is consistent with the ACG clinical guidelines, which recommend triple therapy as first-line treatment in patients who reside in areas with low clarithromycin resistance to HPI and no prior history of macrolide exposure.

The ability to perform *Helicobacter pylori* culture with antimicrobial susceptibility testing in a de minimis number of cases highlighted the possibility of a future study. This finding carries significant importance especially for community-based hospitals with limited resources, where the majority of patients are prescribed initial eradication treatment without microbial culture and antibiotic susceptibility testing following the “test-and-treat” strategy [7]. The implementation of quadruple therapy as the first-line treatment raises concerns about medication costs, insurance coverage, side effects, and dosing. These traditional factors may have a significant impact on patient compliance, with potential implications in the infection prognosis.

Understanding community-specific tendencies in the prevalence and sensitivity of *Helicobacter pylori* is an essential component of successful therapy planning, decreasing rates of HPI and its complications including peptic ulcer and gastric cancer incidences. An individualized and susceptibility-driven approach to the treatment improves its outcomes and minimalizes failures.

## Conclusions

In Metropolitan Hospital, *Helicobacter pylori* resistance to clarithromycin-based triple therapy does not seem to be a common problem when factors other than medication sensitivity are excluded, such as noncompliance with treatment regimens and inappropriate testing for eradication.

Our study showed that selected clarithromycin-based therapies were superior for HPI eradication when compared with non-clarithromycin-based triple therapy. Finally, culture with antimicrobial susceptibility testing was used in a de minimis number of cases, which raises awareness for future study.
